# Transcranial magnetic stimulation-induced global propagation of transient phase resetting associated with directional information flow

**DOI:** 10.3389/fnhum.2014.00173

**Published:** 2014-03-25

**Authors:** Masahiro Kawasaki, Yutaka Uno, Jumpei Mori, Kenji Kobata, Keiichi Kitajo

**Affiliations:** ^1^Department of Intelligent Interaction Technology, Graduate School of Systems and Information Engineering, University of TsukubaTsukuba, Japan; ^2^Rhythm-based Brain Information Processing Unit, RIKEN BSI-TOYOTA Collaboration CenterWako, Japan; ^3^Laboratory for Advanced Brain Signal Processing, RIKEN Brain Science InstituteWako, Japan; ^4^School of Fundamental Science and Technology, Graduate School of Science and Technology, Keio UniversityYokohama, Japan

**Keywords:** transcranial magnetic stimulation, electroencephalogram, synchronization, transfer entropy, information flow, transient phase resetting, oscillations

## Abstract

Electroencephalogram (EEG) phase synchronization analyses can reveal large-scale communication between distant brain areas. However, it is not possible to identify the directional information flow between distant areas using conventional phase synchronization analyses. In the present study, we applied transcranial magnetic stimulation (TMS) to the occipital area in subjects who were resting with their eyes closed, and analyzed the spatial propagation of transient TMS-induced phase resetting by using the transfer entropy (TE), to quantify the causal and directional flow of information. The time-frequency EEG analysis indicated that the theta (5 Hz) phase locking factor (PLF) reached its highest value at the distant area (the motor area in this study), with a time lag that followed the peak of the transient PLF enhancements of the TMS-targeted area at the TMS onset. Phase-preservation index (PPI) analyses demonstrated significant phase resetting at the TMS-targeted area and distant area. Moreover, the TE from the TMS-targeted area to the distant area increased clearly during the delay that followed TMS onset. Interestingly, the time lags were almost coincident between the PLF and TE results (152 vs. 165 ms), which provides strong evidence that the emergence of the delayed PLF reflects the causal information flow. Such tendencies were observed only in the higher-intensity TMS condition, and not in the lower-intensity or sham TMS conditions. Thus, TMS may manipulate large-scale causal relationships between brain areas in an intensity-dependent manner. We demonstrated that single-pulse TMS modulated global phase dynamics and directional information flow among synchronized brain networks. Therefore, our results suggest that single-pulse TMS can manipulate both incoming and outgoing information in the TMS-targeted area associated with functional changes.

## Introduction

Increasing evidence indicates that synchronous neural oscillations play an important role in linking multiple brain regions dynamically and in establishing information transfer among these regions (Engel and Singer, 2001; Varela et al., 2001; Ward, 2003). In general, the stable and constant electroencephalogram (EEG) oscillatory phase differences among distant brain regions reveal global synchronization, whereas EEG amplitude typically reveals the extent of task involvement for a local neural ensemble (i.e., local synchronization) (Fries, 2005; Klimesch et al., 2008). It has been demonstrated in humans that such large-scale phase synchronizations lead to dynamic brain networks that mediate cognitive functions, such as visual awareness (Rodriguez et al., 1999; Cosmelli et al., 2004; Kitajo et al., 2007; Melloni et al., 2007), working memory (Mizuhara and Yamaguchi, 2007; Kawasaki et al., 2010; Kawasaki and Yamaguchi, 2013), and attention (Womelsdorf and Fries, 2007; Doesburg et al., 2008). Although phase synchronization analyses can evaluate the interaction or communication among brain areas, it is difficult to identify the causal relationship, or the directional information flow, among these brain areas. Considering that neurons are typically directional cells, the information flow among brain areas should also be directional.

Transcranial magnetic stimulation (TMS) is an ideal method to examine this issue, as it allows a non-invasive stimulation of the human brain that can perturb EEG oscillations (Massimini et al., 2005; Thut et al., 2005). It has been suggested that single-pulse TMS can induce transient neural oscillations in several frequency bands in different cortical areas of the human brain (Paus et al., 2001; Fuggetta et al., 2005; Van Der Werf and Paus, 2006; Taylor et al., 2008; Rosanova et al., 2009; Thut and Miniussi, 2009; Veniero et al., 2011). Furthermore, some of the aforementioned studies have suggested that TMS-induced oscillations reflect phase resetting in ongoing cortical oscillations. To our knowledge, however, almost no study has estimated quantitatively TMS-induced phase resetting, although a recent study has addressed the signal transmission of TMS-modulated EEG phase dynamics (Casali et al., 2010). Moreover, previous studies using event-related brain potential (ERP) analyses have reported that TMS-induced responses propagate globally among distant brain areas (Ilmoniemi et al., 1997; Massimini et al., 2005; Morishima et al., 2009). These findings indicate that it is possible to investigate global frequency-specific phase dynamics by applying TMS while recording EEG activity. We investigated transient phase resetting in the TMS-targeted area and distant areas, their time-course relationships, and directional information flow among the brain areas.

Here, we used an information theoretic approach to test our working hypothesis. Transfer entropy (TE), which is an information theory measure that evaluates directional information transfer between 2 systems (Schreiber, 2000; Kaiser and Schreiber, 2002; Vicente et al., 2011), was used to evaluate the causal information flow between non-linear oscillators in the brain. TE was selected for this analysis because it does not require a model of interaction, and it is not limited to linearity and stationarity, unlike structural equation modeling (Bullmore et al., 2000), dynamic causal modeling (Friston et al., 2003), and Granger causality (Brovelli et al., 2004; Roebroeck et al., 2005) in functional magnetic resonance imaging (fMRI) analyses. In fact, TE was used to show causalities between non-linear biological signals, such as heart and respiration rates (Schreiber, 2000), and auditory cortical neurons (Gourevitch and Eggermont, 2007). We examined TMS-induced global propagations of phase resetting and used TE to quantify the causal and directional information flow among human brain regions. By calculating the TE from the TMS-targeted visual area to another distant area (i.e., motor area), we estimated directional information flow successfully.

## Materials and methods

### Subjects

Ten healthy right-handed volunteers (2 females and 8 males; mean age, 25.8 ± 2.1 years) participated in this experiment. The subjects reported via subjective questionnaires on having normal visual acuity (with or without correction), hearing, and motor abilities. All subjects gave written informed consent prior to participation in this study. The study was approved by the RIKEN Ethics Committee (in accordance with the Declaration of Helsinki). The data obtained from 1 female subject were excluded from the statistical analysis because of the insufficient amount of significant EEG data.

### TMS

While subjects sat in a relaxed position and rested with their eyes closed, single-pulse TMS was delivered to the visual cortex at intervals ranging between 2.5 and 3.5 s. TMS was delivered through a figure-of-eight coil with a 70-mm wing diameter that was connected to a biphasic stimulator (Magstim Rapid, Magstim Company Ltd., UK). To fix the coil at the same position and direction throughout each session, we used the flexible arm of a camera stand. Prior to performing the experiments, we determined the motor threshold (MT) of each subject by applying single TMS pulses over the left motor cortex and recording the intensity at which a single pulse evoked a minimally perceptible movement of the right index finger. When delivering TMS stimulation during the experiment, we fixed the TMS coil over the occipital pole with the handle oriented upward. Under the sham TMS condition, TMS pulses were delivered at a location 15 cm from the top of the head.

### Experimental procedure

Subjects completed 3 sessions in a counter-balanced order. In 2 sessions, TMS targeting the visual cortex (Oz) was delivered at either 95% MT (higher-intensity TMS) or 50% MT (lower-intensity TMS), and in 1 session, subjects underwent a sham-TMS condition at 50% MT. Each session consisted of 50 TMS applications. Throughout each session (duration, 2.5 min), subjects were required to sit in a chair, keep their eyes closed, and maintain their head position within a chin rest. TMS sessions were conducted in a dim electronic- and sound-shielded room. Subjects wore earplugs to help attenuate the effects of TMS-related auditory noises. Furthermore, to confirm the arousal, subjects were asked to respond by pressing a keyboard button by their right index finger when they sensed a white flashed square (visual angle, 1° × 1°; color, [r, g, b] = [255, 255, 255]; luminance, 60 cd/m^2^) that was presented intermittently on a 24 in computer display (ProLite E2410HDS, Iiyama, Japan) between TMS intervals.

### EEG recordings and analyses

EEG was recorded continuously from 67 scalp electrodes (Ag/AgCl) embedded in a TMS-compatible electrode cap (EasyCap; EASYCAP Gmbh, Germany), and in accordance with the placement of the international 10/10 system. EEG signals were referenced digitally to the averaged recordings from the right and left earlobes. Electrode impedance was maintained below 10 kΩ. Electrooculography (EOG) was recorded from electrodes that were placed above and below the left eye, to monitor eye blinks or vertical eye movements. EOG electrodes placed 1 cm lateral from the right and left eyes monitored horizontal eye movements. The EEG and EOG signals were amplified using a BrainAmp MR+ apparatus (Brain Products, Germany). The sampling rate was 1000 Hz. In accordance with a previous study (Sekiguchi et al., 2011), we rearranged the lead wires relative to the coil orientation, to reduce TMS-induced artifacts.

EEG data were preprocessed by first segmenting the EEG data into 5-s epochs (with 3-s pre-TMS and 2-s post-TMS periods; 5000 time points in total). We removed the EEG data points that were affected by TMS artifacts (from −1 to 7 ms from TMS onset) using linear interpolation. The duration of artifacts was consistent with a previous study (Veniero et al., 2009). The EEG data were 0.1 Hz high-pass filtered. Next, epochs containing artifacts caused by blinks or eye movements were detected from the EOG and EEG data using an amplitude criterion (±150 μV) and were excluded from further analysis. Finally, after the 47 Hz low-pass filter, to identify the cortical activity with reduced effects of volume conduction, we applied current source density transformation to the voltage distribution on the surface of the scalp using the spherical Laplace operator (Perrin et al., 1989; Kayser and Tenke, 2006).

To identify the time-frequency phases, we applied wavelet transforms using Morlet's wavelet function (Tallon-Baudry et al., 1996). We used Morlet's wavelets for the high time and frequency resolutions, which allowed us to observe transitions in both the low and high frequency oscillations better (Herrmann et al., 2005). The phase for each time point in each TMS application was the arctangent of the results of the convolution of the original EEG signal *s*(*t*) with a complex Morlet's wavelet function *w*(*t*, *f*):

$$w(t,f)=\sqrt{f}\exp\text{​}\left(-\frac{t^{2}}{2\sigma_{t}^{2}}\right)\exp\text{​}\left(i2\pi ft\right)$$

where σ_*t*_ is a standard deviation of the Gaussian window. The wavelet used here was roughly characterized by the number of cycles *n*_*co*_ within a 6σ_*t*_ interval (Lachaux et al., 2000), which contains about 99.7% of the power of the Gaussian window. We chose *n*_*co*_ = 3 (= 6*f*σ_*t*_), with *f* ranging from 2 to 40 Hz in 1-Hz steps.

### PLF

TMS-evoked phase resetting was calculated using phase locking factors (PLF; (Tallon-Baudry et al., 1996)) at each electrode (*ch*), time point (*t*), and frequency (*f*) as follows.

$$PLF(t,f,ch)=\frac{1}{N}\left|\sum_{n=1}^{N}\exp\text{​}\left(i\varphi (t,f,ch,n)\right)\right|$$

where φ is the instantaneous phase of EEG data and *N* is the total number of epochs included in the calculation. Using the averaged baseline PLF (PLF_*b*_; −1000 to −500 ms from TMS onset), a standardized PLF (PLF_*z*_) was calculated to reduce the formula's sampling number bias for epochs:

$$PLF_{z}(t,f,ch)=\frac{\text{PLF}(t,f,ch)-\bar{{\text{PLF}}_{b}(t,f,ch)}}{\sigma ({\text{PLF}}_{b}(t,f,ch))}.$$

We tested the statistical significance of the difference between PLFz around the TMS application and pre-TMS periods averaged across subjects. Specifically, we obtained a pre-TMS PLF_*z*_ distribution in which we computed PLF_*z*_ from 200 time points selected randomly in pre-TMS periods (−1700 to −500 ms). Subsequently, we tested whether the mean PLF_*z*_ around TMS was higher (or smaller) than the upper (or lower) limit of the 99% confidence interval of the pre-TMS PLF_*z*_ distributions.

### ZPPI

To confirm the PLFz results, we also analyzed another phase resetting measure, the phase-preservation index (PPI) (Mazaheri and Jensen, 2006). The PPI quantifies the consistency in phase stability as a function of time over epochs taking a value between 0 and 1 for each time point (*t*), frequency (*f*), reference time point (*t*_*ref*_), and electrode (*ch*) as follows.

$$\begin{array}{l} \text{PPI}(t,f,t_{ref},ch)=\frac{1}{N}|\sum_{n=1}^{N}\exp\{i(\varphi (t,f,ch,n)\\ \;-\varphi (t_{ref},f,ch,n))\}| \end{array}$$

We tested the statistical significance of the difference between the decay time of PPI around TMS application and pre-TMS periods averaged across subjects. The averaged PPI is more strongly biased by the results of subjects whose number of trials is small (i.e., bad signal/noise ratio) because PPI increases as the number of trials decreases. To decrease the effect of this bias, the PPI was transformed to Rayleigh's *Z*-value using the formula ZPPI = *n* × PPI^2^, where *n* is the number of trials for each subject. Then we averaged ZPPI across subjects (Fisher, 1993; Mazaheri and Jensen, 2006).

First, we obtained 3000 ZPPI for the phase data that were shuffled randomly in time from pre-TMS periods (−1700 to −1000 ms). This procedure provides a ZPPI estimate with no temporal correlations for each subject. We computed the critical value as the upper 5% limit of the null distribution. Next, we assessed the decay time defined as the interval from a reference point to the time point where ZPPI became lower than the critical value.

To detect TMS-induced phase resetting by the ZPPI, the reference time point has to be close enough to TMS onset because ZPPI decays to the critical value around 300 ms even in the pre-TMS baseline periods. If it is too close, however, TMS causes a biased phase distribution at the reference point, which renders it hard to detect TMS-induced phase resetting by the ZPPI. Therefore, we set the reference time point at −300 and −200 ms for electrodes Oz and C3, respectively. Indeed, the phase distributions were not biased because PLFz were not significantly high at the reference points (Figures 2A–C).

Finally, we assessed if the decay time of ZPPI around TMS application was faster than the decay time of pre-TMS ZPPI. Specifically, we estimated the 95% confidence intervals of 200 pre-TMS ZPPI curves (and decay time) averaged across subjects, which were computed for pre-TMS periods by setting the reference points randomly between −1700 and −1000 ms (non-shuffled data), and compared them with the subject-averaged ZPPI curve (and decay time) around TMS application.

### Transfer entropy

To estimate directional information flow among brain regions, we used TE, which is an information theory measure developed by Schreiber (2000). TE can quantify the directional information flow between 2 systems *X* and *Y* by quantifying how the future state of *X* is determined by the current states of *X* and *Y*.

To compute TE, entropy rate (*h*_1_) was first calculated using the current observation values *x*_*t*_ and *y*_*t*_ and the time-shifted (τ) observation value *x*_*t* + τ_ as follows.

$$h_{1}=-\sum_{x_{t+\tau },x_{t},y_{t}}p(x_{t+\tau },x_{t},y_{t})\log_{2}p(x_{t+\tau }|x_{t},y_{t}),$$

where *p*(*x*|*y*) denotes the conditional probability and *p(x, y)* denotes the joint probability. If we assume that the 2 systems are independent, the time-shifted observation value (*x*_*t* + τ_) of system *X* is independent of the current observation value of the other electrode, *y*_*t*_. Therefore, the entropy rate (*h*_2_) is defined as:

$$h_{2}=-\sum_{x_{t+\tau },x_{t},y_{t}}p(x_{t+\tau },x_{t},y_{t})\log_{2}p(x_{t+\tau }|x_{t}).$$

The TE (*TE*) from system *Y* to *X* is defined as the difference between *h*_1_ and *h*_2_, as follows.

$$\begin{array}{l} TE_{Y\to X}=h_{2}-h_{1}\\ \;=\sum_{x_{t+\tau },x_{t},y_{t}}p(x_{t+\tau },x_{t},y_{t})\log_{2}\left(\frac{p(x_{t+\tau }|x_{t},y_{t})}{p(x_{t+\tau }|x_{t})}\right) \end{array}$$

Since the formula is not symmetric, we can estimate the information flow between the 2 systems separately for both directions. More specifically, the *TE* from system *X* to *Y* is obtained by,

$$TE_{X\to Y}=\sum_{y_{t+\tau },y_{t},x_{t}}p(y_{t+\tau },y_{t},x_{t})\log_{2}\left(\frac{p(y_{t+\tau }|x_{t},y_{t})}{p(y_{t+\tau }|y_{t})}\right).$$

We used the instantaneous phases of CSD signals from 2 electrodes (i.e., the C3 electrode as the left motor area and the Oz electrode as the visual area) as the observation values of 2 systems, and then estimated the TE between the 2 signals.

A straightforward approach for estimating TE is to divide the state space into bins of a given width and construct multidimensional histograms from the data, to evaluate the probability density (Schreiber, 2000; Vicente et al., 2011). However, the arbitrary bin size often biases the estimate when there is a limited number of data points, which are too sparse in the state space. Non-parametric estimation using kernel techniques is a useful alternative to binning a distribution (Silverman, 1981, 1986; Kaiser and Schreiber, 2002). Therefore, we estimated multidimensional probability density functions using the kernel density estimation method (Silverman, 1981, 1986), rather than using probabilities estimated by empirical histograms. As phase is a circular (i.e., π = −π) measure, we used a von Mises distribution, which is a continuous probability distribution on the circle, as a kernel density function. The kernel bandwidth (κ = 8) was optimized using the least-squares cross-validation method (Sain et al., 1994).

Next, we used the estimated multidimensional probability density function to compute TE, as follows.

$$TE_{Y\to X}=∭p(x_{t+\tau },x_{t},y_{t})\log_{2}\left(\frac{p(x_{t+\tau }|x_{t},y_{t})}{p(x_{t+\tau }|x_{t})}\right)dx_{t}dx_{t+\tau }dy_{t}.$$

The integral was numerically estimated via a 20-point Gauss–Legendre quadrature.

We used phase data extracted in a 200-ms period around the time of a TMS pulse (pre-100 ms, post-100 ms) as the current observation values *x*_*t*_, *y*_*t*_, and phase data from a time-lagged 200-ms period as the corresponding observation values *x*_*t* + τ_. We estimated the TE for each subject as the function of the time lag (τ) ranging from 0 to 600 ms, in 5-ms steps. We tested the statistical significance of the difference between TE around the TMS application and pre-TMS periods averaged across subjects. We obtained a 200-ms pre-TMS TE distribution in which we computed TE from 200 sets of randomly selected consecutive 200-ms pre-TMS periods (−1700 to −500 ms). More specifically, we tested whether the mean TE around TMS was higher (or smaller) than the upper (or lower) limit of the 99% confidence interval of the pre-TMS TE distributions.

### Additional experiments

We conducted additional experiments to address (1) the effects of the TMS click sound on the EEG (Nikouline et al., 1999), (2) the influences of the sham TMS intensity (50% MT), and (3) the signal/noise ratio (i.e., effects of the number of trials).

We included 12 healthy right-handed volunteers (6 females and 6 males; mean age, 26.3 ± 6.1 years) in this experiment and used the same subjective questionnaires used in the previous study. The additional experiments were approved by the institutional ethics committee. The data obtained from 2 subjects were excluded from the statistical analysis because the TMS intensities were not large enough to evoke responses.

The additional experiments included 1 visual TMS condition and 2 sham TMS conditions. The experimental paradigm, environment, and equipment were similar to those for the previous experiments, except for the following points. First, subjects wore in-ear headphones with earmuffs and listened to a masking white noise. The noise was adjusted so that the subjects could not hear the TMS coil click during the TMS experiments (Paus et al., 2001; Fuggetta et al., 2005; Massimini et al., 2005). Second, the TMS intensity was set to 95% or 50% MT in all 3 conditions. Third, we placed a thin layer of plastic foam between the scalp and the coil when the coil was positioned over the visual area to attenuate conduction of the TMS click through the bone (Paus et al., 2001; Massimini et al., 2005; Van Der Werf and Paus, 2006; Rosanova et al., 2009; Casali et al., 2010; Mäki and Ilmoniemi, 2010; Ter Braack et al., 2013). Fourth, 100 trials were completed in all conditions.

The sham TMS condition was divided into 2 types. In the first type (sham1), the TMS coil handle was oriented rightward with the handle axis rotated 90 degrees so that only one wing of the figure-of-eight coil was oriented to the scalp. A 3.6-cm plastic cube with a thin layer of plastic form was used as a spacer that was placed between the occipital pole and the coil wing (Esser et al., 2006). In the second type (sham2), the TMS pulses were delivered at a location 15 cm from the top of head.

The statistical analyses were similar to those performed in the previous experiments, except that the reference time point in the PPI analyses was −300 ms from the TMS onset for electrode C3, whereas the previous experiments used −200 ms. We added 100 ms because the rise time of the C3 PLF_z_ in the additional experiments was faster than that in the previous experiments. Representative EEG signals and averaged TMS evoked potentials for the main and additional experiments are shown in Figures S1 and S2.

## Results

### PLF_*z*_ and ZPPI results

**Figure 1:**
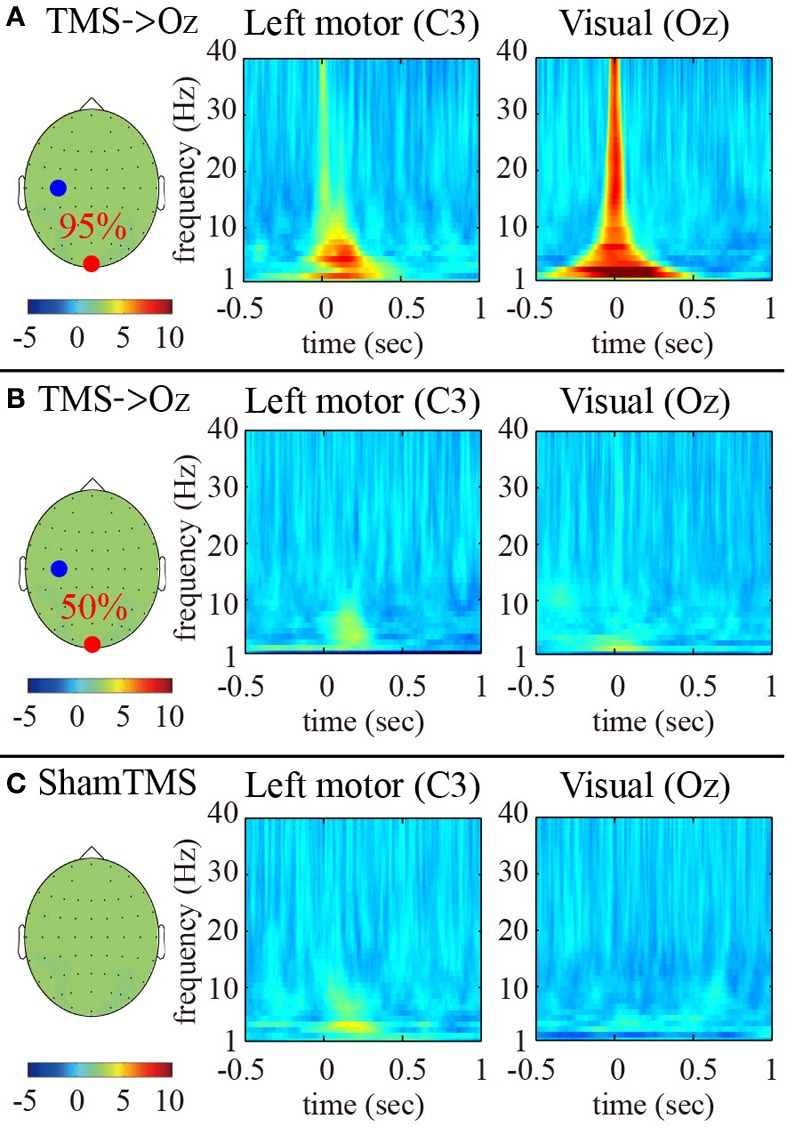
**Subject-averaged (*N* = 9) time-frequency *PLFz* of the C3 (left) and Oz (right) electrodes at the onset of TMS applications under the 95% MT TMS (A), 50% MT TMS (B), and sham-TMS (C) conditions**. The left topographies show the C3 (blue) and Oz (red) electrodes on the recording montage.

The time-frequency EEG results showed transient enhancements of PLF_*z*_ ranging from 2 to 40 Hz at the TMS-targeted electrode at the times at which TMS was delivered under the higher-intensity (95% MT) TMS condition (Figure 1A). These enhancements were observed ahead of the TMS onsets because of the wavelet time resolution. In particular, the low frequency PLF_*z*_, especially theta (4–8 Hz) oscillations, increased from the TMS onset at both the TMS target locations (i.e., Oz) and the distant brain areas (e.g., C3; the electrode showing the maximum PLF_*z*_). The instant amplifications of PLF_*z*_ at the TMS target electrodes increased significantly as TMS increased from lower (50% MT) to higher intensities (Figures 1A,B; false-discovery rate corrected *P* < 0.01; Wilcoxon sign rank test).

**Figure 2:**
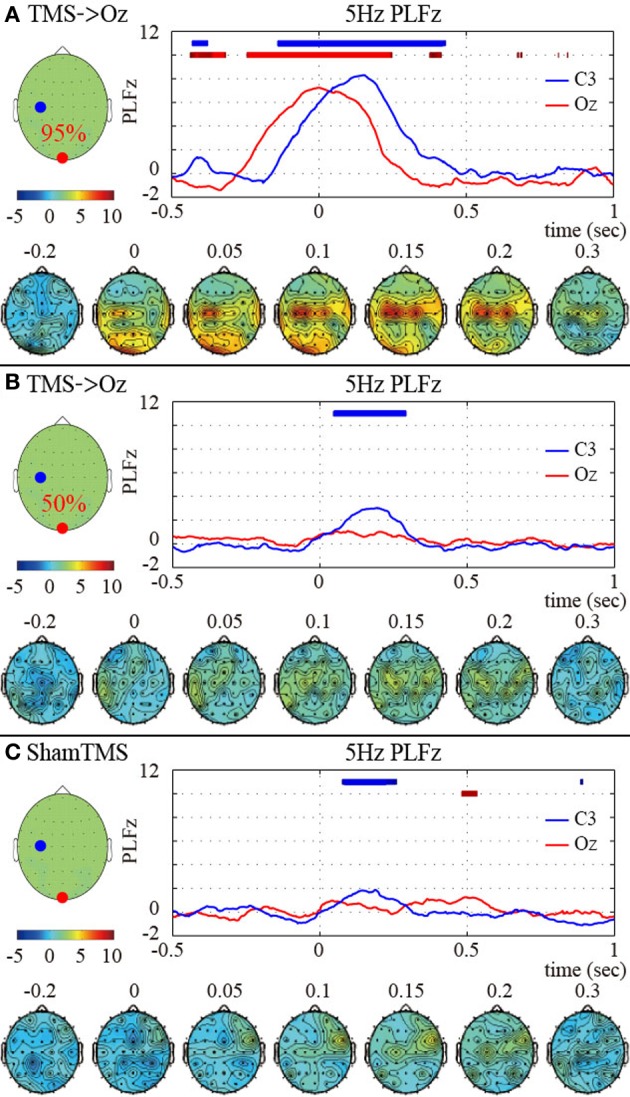
**Subject-averaged (*N* = 9) time course of 5 Hz *PLFz* (top) and their topographies (bottom) of the C3 (blue) and Oz (red) electrodes at the onset of TMS applications under the 95% MT TMS (A), 50% MT TMS (B), and sham-TMS (C) conditions**. The thick lines in the upper part of line graphs indicate the time periods in which *PLFz* was significantly higher than the pre-TMS periods at the C3 (blue) and Oz (red) electrodes (*P* < 0.01). The left topographies show the C3 (blue) and Oz (red) electrodes on the recording montage.

We observed global phase resetting in the distant brain regions. With higher-intensity TMS, transient phase resetting of the theta (frequency-measuring peak PLF_*z*_; 5 Hz) oscillations was transmitted from the visual areas to the motor areas (in particular the left motor area) (Figure 2A). The TMS-enhanced theta PLF_*z*_ was significantly higher than those of the pre-TMS periods in both the visual and motor areas. In addition, the left motor electrode showed the highest theta PLF_*z*_ from approximately 152 ms after TMS onset, whereas PLF_*z*_ at the visual electrode reached peak factors at the time TMS was applied. Such observation of phase resets at the distant electrode decreased and disappeared with lower-intensity TMS and with sham TMS (Figures 2B,C).

**Figure 3:**
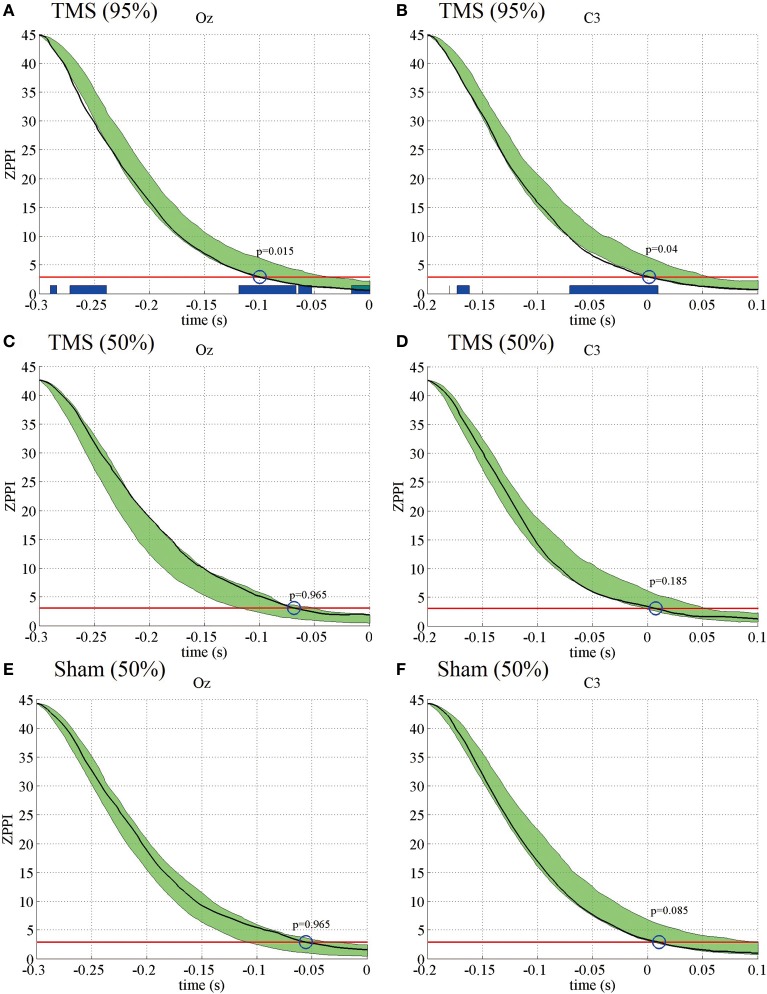
**Subject-averaged (*N* = 9) PPI for the electrodes Oz and C3 under the 95% MT TMS (A,B), 50% MT TMS (C,D), and Sham-TMS conditions (E,F)**. The black thick lines indicate PPI around TMS applications (reference time point at −300 and −200 ms for the electrodes Oz and C3, respectively). The red lines indicate the critical values defined as the upper 5% limit of the null distribution. The greenish areas indicate the 95% confidence intervals of pre-TMS PPI. Using the 95% confidence intervals, we assessed if the decay time of PPI to the critical value around TMS application was significantly shorter than the decay time of pre-TMS PPI (blue circles and *P*-values). The blue thick lines in the lower part of graphs indicate the time periods in which PPI decayed significantly faster than pre-TMS PPI.

Next, we analyzed if the TMS-induced increase in PLF_*z*_ was associated with changes in another phase-resetting measure, ZPPI. Figure 3 shows the ZPPI computed for 5-Hz phase at the TMS target locations (Oz) (Figure 3A) and the distant brain area (C3) (Figure 3B) averaged across subjects under the higher-intensity (95% MT) TMS condition. ZPPI around TMS application (thick black line), which was computed using the reference time point of −300 ms, showed a significantly shorter decay time (*P* < 0.05) to the critical value (red line) than did the pre-TMS ZPPI curves (−1700 to −500 ms) (95% confidence intervals, greenish areas in Figure 3) for the Oz electrode. ZPPI around TMS application, which was computed using the reference time point of −200 ms for the C3 electrode, was significantly shorter (*P* < 0.05) in decay time to the critical value (red line), than was the pre-TMS ZPPI. Figures 3C,D show the results obtained for the lower-intensity TMS (50% MT) condition. ZPPI around TMS application was not significantly different from ZPPI of pre-TMS periods for both the Oz and C3 electrodes. In the sham condition, we did not observe any significant changes in ZPPI around sham TMS application for both the Oz and C3 electrodes compared with pre-TMS periods (Figures 3E,F).

These results indicate that the TMS-induced increase in PLF_*z*_ is accompanied by a significantly shorter decay time in ZPPI compared with pre-TMS periods more prominently in the higher-intensity TMS (95% MT) condition.

### Transfer entropy results

**Figure 4:**
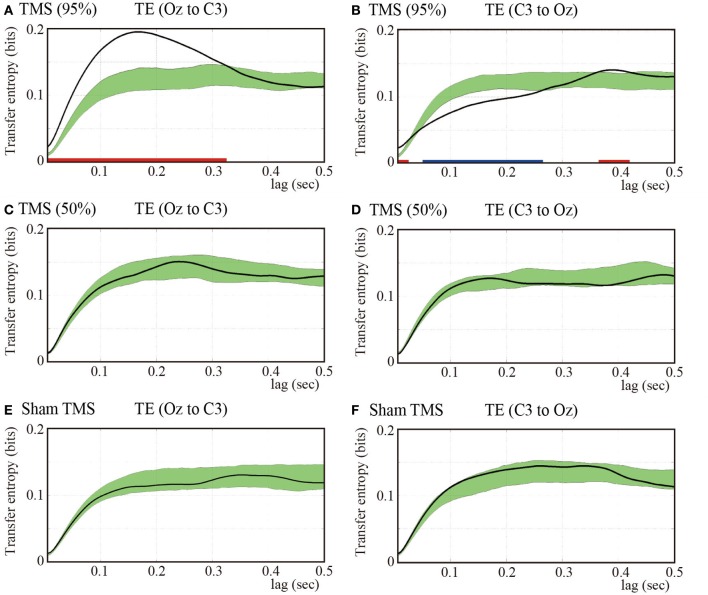
**Subject-averaged (*N* = 9) TE as a function of time lag**. TE (Oz to C3) **(A)** and TE (C3 to Oz) **(B)** under the 95% MT TMS conditions. Greenish areas indicate the 99% confidence intervals calculated using these pre-TMS periods. The red and blue thick lines in the lower part of line graphs indicate the time periods in which TE was significantly higher than the pre-TMS TE (*P* < 0.01). **(C,D)** TE (Oz to C3) and TE (C3 to Oz) under the 50% MT TMS condition. **(E,F)** TE (Oz to C3) and TE (C3 to Oz) under the sham-TMS condition.

Considering that we found the most prominent time-delayed TMS-induced phase resetting from Oz to C3 at 5 Hz, we evaluated the information transfer between electrodes Oz and C3 by computing TE for 5-Hz phase signals. Figure 4A demonstrates mean TE (Oz to C3) as a function of TE time lag with the higher-intensity TMS. In the higher-intensity TMS condition, we observed a prominent peak in the subject-averaged TE (Oz to C3) at a 165-ms lag. We also computed pre-TMS TE from 200-ms periods selected randomly between −1700 and −500 ms pre-TMS, which is a stable period that precedes TMS. Using these pre-TMS periods, we computed the 99% confidence intervals (greenish areas in Figure 4). We observed that the pre-TMS periods were not affected by the previous TMS (i.e., the minimum time interval was 2500 ms) or the PLF_*z*_ enhancements preceding the TMS in that epoch (see Figure 1). TE around TMS application (Oz to C3) was significantly higher than the pre-TMS TE (Oz to C3) with a time lag between 5 and 325 ms in the higher-intensity TMS condition. In addition, we observed a baseline information flow between Oz and C3 in pre-TMS data. These results suggest the existence of information flow from TMS-targeted visual areas (Oz) to motor areas (C3), which was enhanced by single-pulse TMS maximized at a 165-ms lag.

In contrast, we found a significantly lower TE around the time of TMS application (C3 to Oz) compared with the pre-TMS TE with a lag (C3 to Oz) between 50 and 265 ms in the higher-intensity TMS condition. These results indicate that the information flow from C3 to Oz around the time of TMS application was suppressed within this time-lag range. Moreover, TE (C3 to Oz) showed a later peak at a time lag of approximately 400 ms (Figure 4B). The peak of TE around the time of TMS application was significantly different from TE for the pre-TMS periods.

Figures 4C,D show the results obtained for the lower-intensity TMS (50% MT) condition. We observed a less prominent and later peak for TE (Oz to C3) compared with what was observed for higher-intensity TMS. TE (Oz to C3) around the time of TMS application was not significantly different from pre-TMS TE (Oz to C3). Moreover, TE (C3 to Oz) was not significantly different from TE (C3 to Oz) of the pre-TMS periods. In the sham condition, we did not observe any significant changes in TE around TMS application for either direction (i.e., Oz to C3 and C3 to Oz) from pre-TMS periods (Figures 4E,F).

The TE results showed that TMS can enhance directional information flow from the TMS-targeted visual area to motor areas with higher-intensity TMS, which is consistent with the PLF_*z*_ and ZPPI results that indicated prominent propagation of transient phase resetting from the TMS-targeted visual areas to the motor areas.

### ZPPI results of the additional experiments: PLF_*z*_, ZPPI, and TE

**Figure 5:**
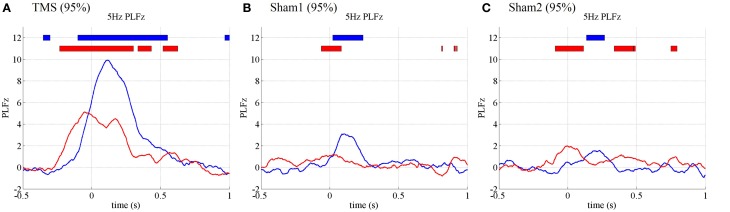
**Subject-averaged (*N* = 10) time course of 5 Hz *PLFz* at the onset of TMS applications under the 95% MT TMS (A), 95% MT sham1 TMS (B), and 95% MT sham2 TMS (C) conditions in the additional experiments**. The thick lines in the upper part of line graphs indicate the time periods in which *PLFz* was significantly higher than the pre-TMS periods at the C3 (blue) and Oz (red) electrodes (*P* < 0.01).

**Figure 6:**
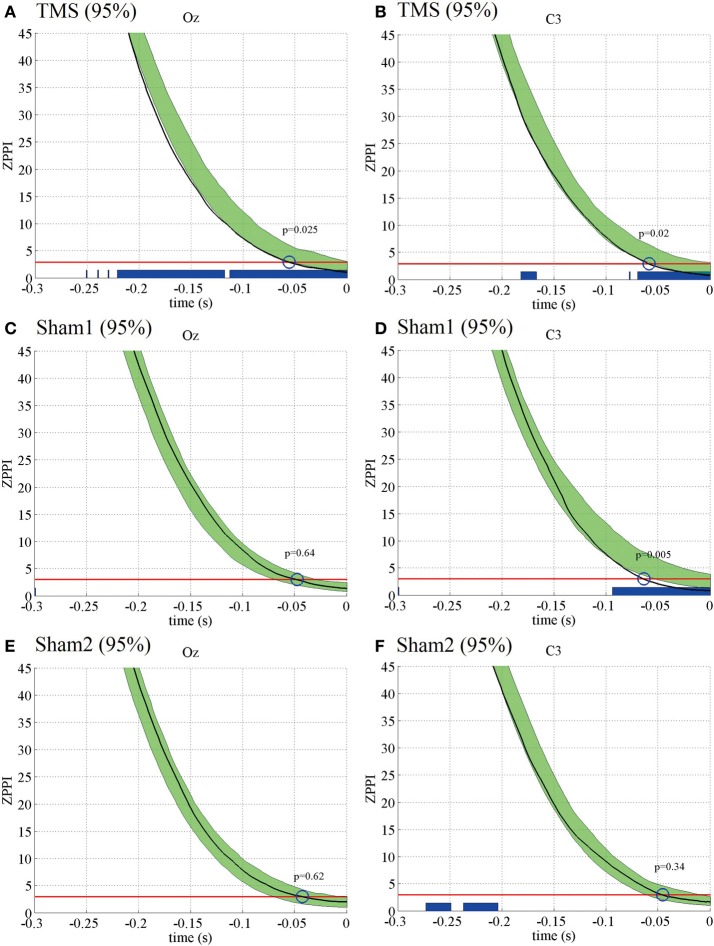
**Subject-averaged (*N* = 10) ZPPI for the electrodes Oz and C3 under the 95% MT TMS (A,B), 95% MT sham1 TMS (C,D), and 95% MT sham2 TMS (E,F) conditions in the additional experiments**. The black thick lines indicate ZPPI around TMS applications (reference time point at −300 and −200 ms for the electrodes Oz and C3, respectively). The red lines indicate the critical values defined as the upper 5% limit of the null distribution. The greenish areas indicate the 95% confidence intervals of pre-TMS ZPPI. Using the 95% confidence intervals, we assessed if the decay time of ZPPI to the critical value around TMS application was significantly shorter than the decay time of pre-TMS ZPPI (blue circles and *P*-values). The blue thick lines in the lower part of graphs indicate the time periods in which ZPPI decayed significantly faster than pre-TMS ZPPI.

**Figure 7:**
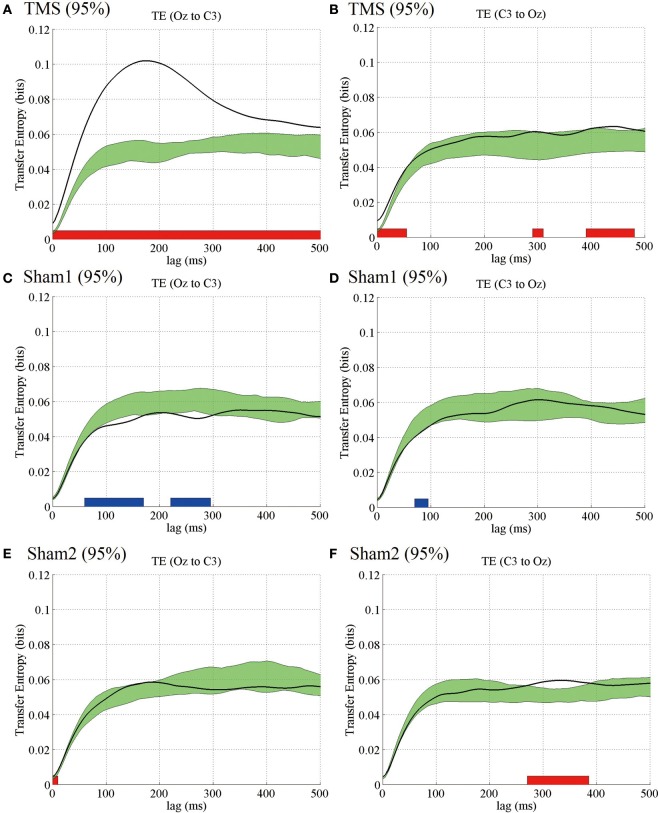
**Subject-averaged (*N* = 9) TE as a function of time lag**. TE (Oz to C3) **(A)** and TE (C3 to Oz) **(B)** under the 95% MT TMS conditions. Greenish areas indicate the 99% confidence intervals calculated using these pre-TMS periods. The red and blue thick lines in the lower part of line graphs indicate the time periods in which TE was significantly higher than the pre-TMS TE (*P* < 0.01). **(C,D)** TE (Oz to C3) and TE (C3 to Oz) under the 95% MT sham1 TMS, and **(E,F)** TE (Oz to C3) and TE (C3 to Oz) under the 95% MT sham2 TMS conditions in the additional experiments.

Figures 5–7 presents the results of the additional experiments. These results include the PLF_*z*_, ZPPI, and TE results in the 5-Hz phases under the visual TMS, as well as the sham1 and sham2 TMS conditions (all TMS intensities were 95% MT).

The PLFz results from the additional experiments replicated those from the previous experiments (Figure 5). In the visual condition (95% MT), the TMS-enhanced 5-Hz PLF_*z*_ was significantly higher than those in the pre-TMS periods for both the visual (Oz) and motor areas (C3). Moreover, the C3 electrode showed the highest theta PLF_*z*_ from 116 ms after TMS onset, whereas the Oz electrode PLF_*z*_ peaked at the time TMS was applied. Such prominent increases in PLFz were not observed in the 2 sham conditions at 95% MT or any condition at 50% MT.

The ZPPI results for the additional experiments also were similar to those for the previous experiments (Figure 6). The ZPPI measured around the time of the TMS application for the Oz and C3 electrodes showed a significantly shorter decay time (*P* < 0.05) to the critical value than did the pre-TMS ZPPI curves in the visual TMS condition at 95% MT. In contrast, the ZPPI measured around the time of the TMS application was not significantly different from the ZPPI of the pre-TMS periods for either the Oz or C3 electrodes in the sham2 condition at 95% MT. However, the ZPPI for the C3 electrode showed a significantly shorter decay time in the sham1 condition at 95% MT. Such a decay in ZPPI was not observed in any condition at 50% MT.

The TE results for the additional experiments also were similar to those for the main experiments. In the visual TMS condition, we observed a prominent peak in the subject-averaged TE (Oz to C3) at 175 ms. The *TE*-values were significantly higher than those for the pre-TMS TE. The TE modulations appearing around the TMS application for the opposite direction (C3 to Oz) in the visual TMS condition and for either direction (i.e., Oz to C3 and C3 to Oz) in the 2 sham conditions were partially significant (see Figure 7).

These results indicate that any artifact produced by the TMS click or a low number of trials should not have affected our finding that TMS can enhance directional information flow from the TMS-targeted visual area to motor areas.

## Discussion

The present study demonstrated that single-pulse TMS can induce a large-scale propagation of transient phase resetting that is accompanied by causal information flow from the TMS-targeted area to distant areas via bottom-up synchrony networks during TMS-induced brain states. Previous TMS–EEG studies have shown either TMS-induced oscillations within local areas (Paus et al., 2001; Fuggetta et al., 2005; Van Der Werf and Paus, 2006; Taylor et al., 2008; Rosanova et al., 2009; Thut and Miniussi, 2009; Veniero et al., 2011) or global propagation of averaged responses (Ilmoniemi et al., 1997; Massimini et al., 2005; Morishima et al., 2009). In addition, the present study revealed several important findings. First, the time-frequency EEG analysis showed that the PLF_*z*_ reached the highest factor at the distant area with a time lag that followed the peak PLF_*z*_ enhancements of the TMS-targeted area. Second, the TMS-induced increase in PLF_*z*_ was accompanied by a significantly shorter decay time in ZPPI compared with pre-TMS periods more prominently in the higher-intensity TMS (95% MT) condition. Third, the TE from the TMS-targeted area to the distant area clearly increased with a time lag from the TMS onset. Interestingly, the averaged time lags were almost coincident between the PLF_*z*_ (152 ms) and TE (165 ms) results. This finding provides strong evidence that the emergence of the delayed phase resetting, which was indicated by the delayed PLF_*z*_ peaks, is associated with the incoming causal information flow from the TMS-targeted visual area to the non-target motor area, as was indicated by TE.

TMS appears to manipulate the oscillatory phase dynamics and causal information flow among large-scale brain networks in a TMS-intensity-dependent manner, as such TMS-induced modulations were observed only in the higher-intensity TMS condition. The slightly increased PLF_*z*_ observed under the sham and low-intensity TMS conditions in the main experiment might not be due to the information transfer from the visual area to the motor area, because the TE was not significantly different from TE observed in the pre-TMS periods. Global oscillatory modulations were found in the theta range (peak frequency, 5 Hz), although TMS consistently induces frequency-specific oscillations in each brain area, such as alpha oscillations in the occipital areas and beta oscillations in the motor areas (Rosanova et al., 2009). Previous findings indicate that the slow-oscillatory (i.e., theta) synchronization plays an important role in linking dynamically brain areas within the resting-state networks (Von Stein and Sarnthein, 2000; Buzsaki and Draguhn, 2004; Jensen and Colgin, 2007). Slow-oscillatory (i.e., theta) synchronization is similar to the global theta phase synchronization observed for several cognitive functions, including working memory (Sauseng et al., 2005; Mizuhara and Yamaguchi, 2007; Klimesch et al., 2008; Kawasaki et al., 2010). Therefore, the PLF_*z*_ patterns of different frequency oscillations in different brain regions, and the relationships between resting and cognitive functions, should be clarified in future studies.

Although the PLF and ZPPI results possibly suggest the macroscopic phase resetting at the EEG level, the findings are not direct evidence for the microscopic phase resetting at the single neuron level. Previous studies argued that such phase modulations could be either related to additive evoked responses or phase resetting (Sauseng et al., 2007; Becker et al., 2008). Moreover, it is not clear whether the macroscopic phase resetting of EEG oscillations reflects the microscopic phase resetting or additive evoked responses at the single neuron level (Telenczuk et al., 2010). Therefore, it is necessary to examine the issues by combining experimental data at different spatial scales using several indices and mathematical modeling.

Moreover, synchronous and oscillatory phenomena in the brain actually show very transient dynamics. We therefore think it is very difficult to identify whether the TMS-induced response observed in the current study were perfect phase resetting or additive evoked responses. We therefore used the phrase “transient phase resetting” in our study. In addition the transient TMS-evoked phase dynamics in the present study are novel and important.

Moreover, it should be noted that artifacts such as volume conduction of TMS-evoked activity do not explain the propagation of transient phase resetting and directional information flow, as there was a time lag between PLF_*z*_ peaks that matched the optimal lag at which the TE from Oz to C3 was maximized. If the global propagation of transient phase resetting and information transfer was a spurious phenomenon caused by volume conduction, then there should be no time lag.

Furthermore, it is possible that the EEG data was slightly affected by indirect TMS effects, such as the air- and bone-conducted sound produced by the TMS coil click (Nikouline et al., 1999) in the main experiments. To reduce such effects, we applied TMS slightly (about 0.5 cm) above the scalp over the occipital area and asked subjects to wear earplugs. Unfortunately, the method did not prevent auditory effects perfectly, because a weak increase in PLFz was observed under the sham-TMS condition, which does not have direct TMS effects on brain activity (Figure 2C). Moreover, the main experiments lacked higher-intensity sham-TMS data (95% MT).

To address these issues, we conducted additional experiments in which the subjects listened to a masking white noise sound with headphones. In addition, we included two sham conditions in which the TMS intensity was 95% MT or the TMS was applied near the visual area. The PLF_*z*_, ZPPI, and TE results for the additional experiments were similar to those for the previous experiments. The increased PLFz, faster ZPPI decay, and TE enhancements from Oz to C3 were observed in only the visual TMS condition but not the sham2 condition (i.e., the TMS pulses were delivered at a location 15 cm from the top of head). These results suggest that our findings were not influenced by auditory evoked potentials evoked by the associated TMS click sound or by the TMS intensity and location in the sham conditions.

Note that the enhancement of the ZPPI decay in the motor area was significant only in the sham1 condition (i.e., with placing a cube with thin layer of plastic foam between the scalp and the rotated coil) in the additional experiment, whereas the enhanced ZPPI decay was not observed in the visual area. Such results might be due to somatosensory stimulation by bone-conducted vibration from the TMS click but not by auditory effects of the TMS click. It is because that the phase resetting was not found in the sham2 condition, although the auditory masking was same between the sham1 and sham2 conditions. However, this possibility did not influence our findings regarding phase resetting in the motor area in the 95% MT visual TMS condition because the phenomenon was commonly found in both the main experiments (without the somatosensory effects) and additional experiments (without the auditory effects).

Our findings suggest the existence of a potential bottom-up network from the sensory input regions to the motor output regions (i.e., the motor areas) through the corticocortical and/or subcortical networks (Ilmoniemi et al., 1997) in the TMS-induced state. We hypothesized that the motor area that is contralateral to the dominant hand appears to be a goal of the TMS-induced brain state network, based on the convergence of transient phase resetting on the left motor areas in the right-handed subjects included in this study. The bottom-up mechanism would be related to a spontaneous preparation of reaction series, such as the task used in this study. For example, we can imagine seeing a visual stimulus, making a decision, and then responding via output from the motor areas.

We asked the subjects to respond to a visual flash (with their eyes-closed), to monitor and keep the arousal level. Thereafter, the visual flashes possibly influence the arousal level and indirectly affect the excitability of the visual cortex, although the visual flashes are assumed to be temporally far from the TMS timing, not to be presented in every TMS interval (10 flashes and 50 TMS applications in each session), and not to affect the TMS-induced response directly.

It has been reported that TMS induces rapid (<50 ms) increases in firing rates in the cat V1 cortex (Moliadze et al., 2003). This should be related to the transient phase resetting of local oscillations in the targeted area. In response to the TMS-induced increase in spike rate, the targeted area can subsequently increase the strength of its outputs to other areas. This might account for the enhanced bottom-up information flow observed in the current study.

TE is a model-free measure of information flow without the assumption of linearity and stationarity (Schreiber, 2000; Kaiser and Schreiber, 2002; Vicente et al., 2011). TE would be an appropriate information theoretical measure for an exploratory investigation of information flow in complex networks, such as the brain. Our results provide evidence that TE is an appropriate information theoretical measure that evaluates the causal and directional information flow in the brain. Interestingly, it has been shown that Ganger causality and TE are equivalent to Gaussian variables (Barnett et al., 2009). As phase is a circular measure, however, we cannot use Granger causality, which assumes a Gaussian distribution in predicting signals, for detecting causal information flow in phase signals. Therefore, we propose that TE is useful to detect information flow in phase signals, as it evaluates the causal and directional information flow between specific brain areas in synchronous networks. Although we focused our analyses on information transfer between 2 specific brain areas in the current study, future work should analyze information flow more extensively across the entire brain. In fact, TE has been used to detect information flow in complex systems, such as social information flow on the web (Oka and Ikegami, 2012).

The results of the current study indicate the possibility that TE is a quantifiable index of causal information flow among brain networks. Our experimental paradigm had the explicit consequence of information flow by virtue of TMS. In fact, TE indicates that TMS can modulate large-scale causal information flow in the brain, particularly in the case of higher-intensity TMS. Therefore, future studies should examine whether TE reflects causality among brain areas without TMS application.

In conclusion, we demonstrated that single-pulse TMS modulated global phase dynamics and information flow among synchronous networks in the human brain. Our results suggest that single-pulse TMS modulates both incoming and outgoing information flow in the TMS-targeted areas associated with functional changes.

### Conflict of interest statement

The authors declare that the research was conducted in the absence of any commercial or financial relationships that could be construed as a potential conflict of interest.

## Supplementary material

Figure S1 **Representative EEG data from 1 subject in the main experiments under the 95% MT TMS, 50% MT TMS, and 50% sham-TMS conditions**. Upper panels: 20 single-trial EEG signals (dashed lines) for the C3 and Oz electrodes randomly selected from all trials for each TMS condition. 0 ms indicates TMS onset. EEG signals were low cut filtered (30 Hz) by a Butterworth filter. Lower panels: Corresponding averaged TMS evoked potentials (solid lines) for each condition in each subject.

Figure S2 **Representative EEG data from 1 subject in additional experiments under the 95% MT TMS, sham1, and sham2 conditions**. Upper panels: 20 single-trial EEG signals (dashed lines) for the C3 and Oz electrodes randomly selected from all trials for each TMS condition. 0 ms indicates TMS onset. EEG signals were low cut filtered (30 Hz) by a Butterworth filter. Lower panels: Corresponding averaged TMS evoked responses (solid lines) for each condition in each subject.
