# Supplementary figures and images for: Transcranial magnetic stimulation-induced global propagation of transient phase resetting associated with directional information flow
Source: Front Hum Neurosci. 2014 Mar 25;8:173. doi: 10.3389/fnhum.2014.00173 (PMC3971180; doi:10.3389/fnhum.2014.00173)

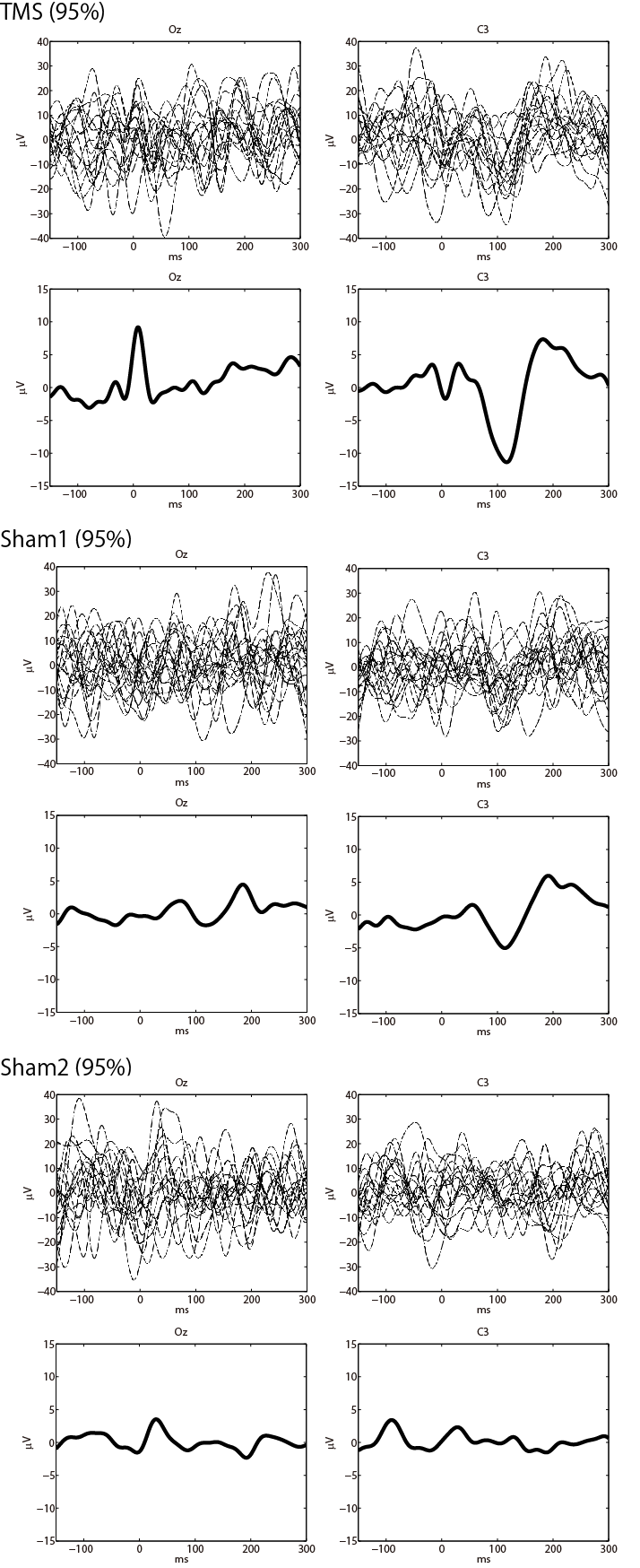

Supplement: Figure S1 — Representative EEG data from 1 subject in the main experiments under the 95% MT TMS, 50% MT TMS, and 50% sham-TMS conditions. Upper panels: 20 single-trial EEG signals (dashed lines) for the C3 and Oz electrodes randomly selected from all trials for each TMS condition. 0 ms indicates TMS onset. EEG signals were low cut filtered (30 Hz) by a Butterworth filter. Lower panels: Corresponding averaged TMS evoked potentials (solid lines) for each condition in each subject. [file Presentation1.ZIP › 59326_Kawasaki_Suppl Figure_2.TIF]

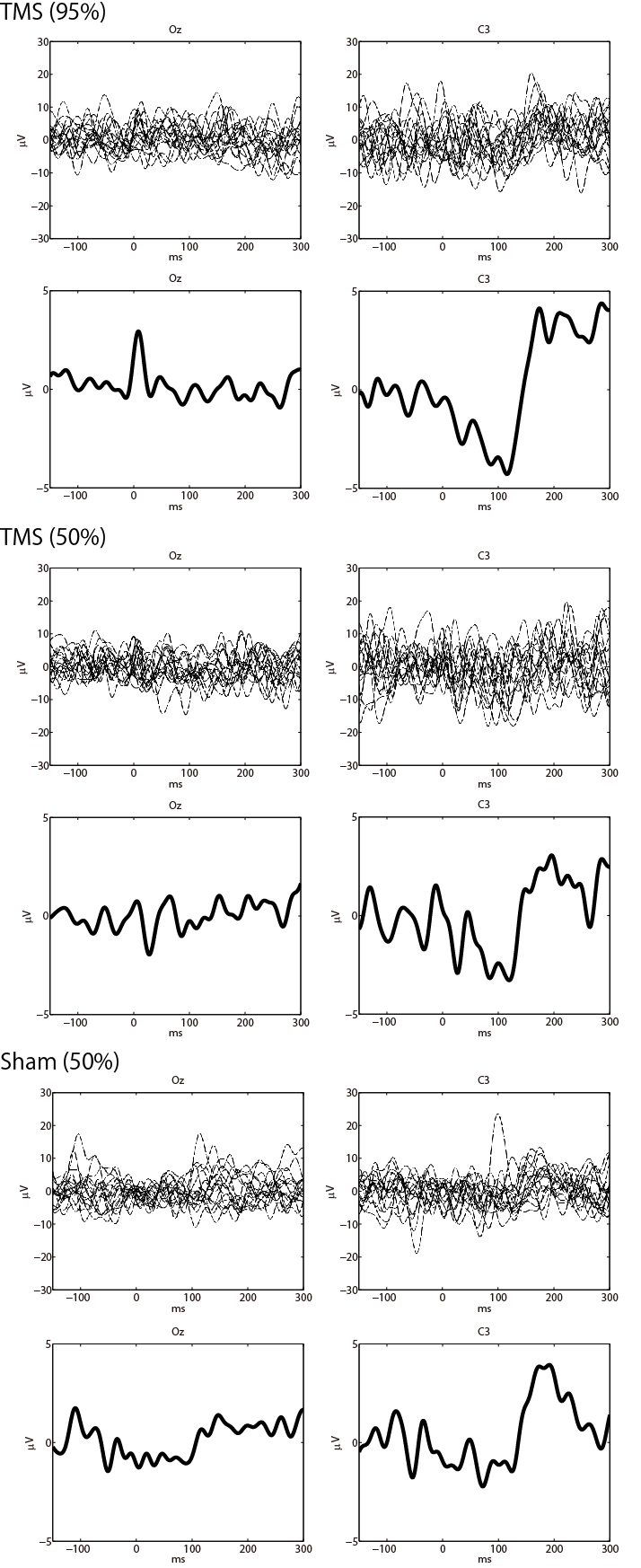

Supplement: Figure S1 — Representative EEG data from 1 subject in the main experiments under the 95% MT TMS, 50% MT TMS, and 50% sham-TMS conditions. Upper panels: 20 single-trial EEG signals (dashed lines) for the C3 and Oz electrodes randomly selected from all trials for each TMS condition. 0 ms indicates TMS onset. EEG signals were low cut filtered (30 Hz) by a Butterworth filter. Lower panels: Corresponding averaged TMS evoked potentials (solid lines) for each condition in each subject. [file Presentation1.ZIP › 59326_Kawasaki_Suppl Figure_1.TIF]
